# Clinical utility of anthropometric parameters in identifying glucose dysregulation in women with polycystic ovary syndrome

**DOI:** 10.3389/fendo.2025.1756679

**Published:** 2026-01-26

**Authors:** Justyna Nowak, Marzena Jabczyk, Jakub Borszcz, Paweł Jagielski, Barbara Zubelewicz-Szkodzińska

**Affiliations:** 1 Department of Metabolic Disease Prevention, Medical University of Silesia, Bytom, Poland; 2 Department of Cardiovascular Disease Prevention, Medical University of Silesia, Bytom, Poland; 3 Department of Nutrition-Related Disease Prevention, Medical University of Silesia, Bytom, Poland; 4 Student Scientific Circle Affiliated of Department of Metabolic Disease Prevention, Faculty of Public Health in Bytom, Medical University of Silesia, Bytom, Poland; 5 Department of Nutrition and Drug Research, Institute of Public Health, Faculty of Health Science, Jagiellonian University Medical Collage, Karków, Poland; 6 Department of Endocrinology, District Hospital, Piekary Śląskie, Poland

**Keywords:** anthropometric indices, cardiometabolic health, insulin resistance, metabolic characteristics, polycystic ovary syndrome

## Abstract

**Background:**

Polycystic ovary syndrome (PCOS) is a common endocrine disorder often associated with disturbances in glucose metabolism and insulin resistance (IR), increasing the risk of type 2 diabetes (T2DM). Standard assessment of glucose dysregulations and IR requires laboratory tests, but simple anthropometric indices, including BMI, WHtR, WHR, VAI, LAP, BAI, BRI and ABSI, may provide non-invasive tools for early risk screening. Their predictive value and optimal cut-off points for detecting glucose dysregulation and IR in PCOS remain unclear.

**Objectives:**

This study aims to evaluate the clinical utility of anthropometric indices in identifying glucose dysregulation in women with PCOS, and to provide prognostic insight with potential cut-off points for these indices.

**Methods:**

This cross-sectional study included 49 women with PCOS according to Rotterdam criteria. Anthropometric measurements (BMI, WC, WHR, WHtR, VAI, BAI, LAP, BRI, ABSI) and fasting biochemical parameters (glucose, insulin) were collected. Correlations between indices and carbohydrate disturbances were assessed using Pearson or Spearman coefficients. The predictive ability of anthropometric indices for glucose dysregulations and IR were evaluated using ROC curve analysis, including AUC, sensitivity, specificity, and optimal cut-off points, while logistic regression quantified the strength of associations.

**Results:**

BMI, WHtR, BAI, VAI, LAP, and BRI were significantly correlated with fasting glucose and insulin levels, indicating a strong link between adiposity and IR in women with PCOS. Among these indices, VAI showed the highest predictive performance for elevated HOMA-IR (AUC = 0.933, cut-off point 0.99; sensitivity 85.7, specificity 90.5%), followed by LAP (AUC = 0.883, cut-off point 27.9) and BMI (AUC = 0.852, cut off point 27 kg/m^2^). WHtR, WC, and BRI also demonstrated significant predictive value (AUCs 0.821-0.831). Logistic regression revealed the strongest associations for BMI ≥27.25 kg/m^2^ and VAI ≥1.07 (OR = 57.0; 95% CI 9.41-345.15; p<0.001), with WC, WHtR, LAP, BAI, and BRI also showed significant predictive value for IR.

**Conclusion:**

Anthropometric indices, particularly VAI, LAP, and BMI, reliably predicts glucose dysregulations and IR in women with PCOS. These simple, non-invasive measurements may serve as useful screening tools for early identifications of glucose dysregulation, aiding risk stratification and guiding further metabolic assessment.

## 1 Introduction

Polycystic ovary syndrome (PCOS) is a common endocrine and metabolic disorder, affecting approximately 10% of women of reproductive age, depending on the diagnostic criteria applied (1, 2). It is a heterogenous disease with diverse clinical manifestation characterized by hyperandrogenism, oligo- or anovulation, and the presence of ovarian morphology. PCOS can manifest in different clinical patterns, based on Rotterdam criteria i.e. hyperandrogenism (clinically or biochemically), oligo-ovulation, and polycystic ovarian morphology (PCO) (1, 3). Several studies have shown that most women with PCOS present an impaired glucose tolerance, insulin resistance (IR) and compensatory hyperinsulinism, abdominal obesity, metabolic disorders, and metabolic syndrome (MetS) (4, 5).

PCOS exhibits usually with persistently elevated gonadotropin-releasing hormone (GnRH) pulsatility, increased luteinizing hormone (LH), and relatively decreased follicle-stimulating hormone (FSH) levels. These alterations in gonadotropin signaling contribute to heightened androgen production and ovulatory dysfunction (6). Hyperglycaemia and hyperinsulinemia enhance ovarian insulin receptor signaling and suppress hepatic sex hormone-binding globulin (SHBG), thereby increasing luteinizing hormone (LH) activity and the free androgen index (FAI), which may contribute to hyperandrogenism (7–9) Additionally, hyperinsulinemia indirectly promotes testosterone production in adipose tissue by modulating lipid metabolism and reducing androgen binding via SHBG (8, 10, 11). Hyperinsulinemia is observed in approximately 65–95% of women with PCOS, affecting most overweight and obese individuals and over half of women of normal weight (10). Metformin or myo-inositol are widely used to improve glucose tolerance and reduce IR in women with PCOS (12, 13) particularly in those with Body Mass Index (BMI) ≥25 (14).

Body weight plays a critical role in both the development and severity of PCOS-related metabolic disturbances. Approximately 80% of women with PCOS have an elevated Body Mass Index (BMI) (15, 16). BMI is a widely used measure for assessing body weight; however, several novel indices incorporating additional anthropometric parameters have been developed. These include waist-to-hip ratio (WHR), waist-to-height ratio (WHtR), Visceral Adiposity Index (VAI), Body Adiposity Index (BAI), Lipid Accumulation Product (LAP), Body Roundness Index (BRI), and A Body Shape Index (ABSI), which may serve as markers of adipose tissue dysfunction in women with PCOS. Notably, BMI, BRI and VAI have been positively associated with insulin resistance (17, 18). Women with PCOS are at an increased cardiometabolic risk, with a higher likelihood of developing type 2 diabetes mellitus (T2DM) and MetS (19–21). Those with additional risk factors, such as IR and/or hyperandrogenism, are particularly susceptible to cardiovascular complications, independent of obesity (11, 22, 23). Early detection of glucose disturbances and IR in women with PCOS is crucial to reduce the cardiometabolic complications. While the hyperinsulinemic-euglycemic clamp is the gold standard in diagnostic IR, it is impractical in routine clinical settings. The homeostatic model assessment of IR (HOMA-IR) provides practical alternative (24). Although fasting glucose and fasting insulin both require blood sampling, only fasting glucose is part of routine clinical screening in primary care, whereas insulin assessment is limited to specialized settings. Thus, indices incorporating fasting insulin (e.g. HOMA-IR) are useful diagnostically, but their clinical utility in population-wide screening is limited (14). In contrast, anthropometric indices combined with fasting glucose provide an accessible alternative for preliminary identification of glucose dysregulation, particularly when supported by clinically defined cut-off values (25).

Therefore, the aim of this study was to establish clinical utility and optimal cut-off points for anthropometric indices for glucose dysregulation in women with PCOS.

## 2 Materials and methods

### 2.1 Study participants

Research was carried out at the Endocrinology Unit of Piekary Medical Centre, located within St. Luke’s Municipal Hospital in Piekary Śląskie, Poland. The study cohort included women with diagnosis of PCOS who met the 2004 Rotterdam criteria. These criteria required the presence of at least two of the following three abnormalities: 1. Polycystic ovarian morphology on ultrasound, defined as a ≥12 follicles measuring 2–9 mm in each ovary and/or an increased ovarian volume exceeding 10 mL, 2. Clinical and/or biochemical evidence of hyperandrogenism (serum testosterone concentration >70 ng/dL, 3. Oligomenorrhea or amenorrhea (17).

Hyperandrogenism was diagnosed based on elevated androgen levels (testosterone and/or androstenedione) and/or the presence of clinical features such as excessive body and facial hair (hirsutism) and acne.

Transvaginal ultrasonography was performed using a 6–12 MHz transducer, which provides optimal resolution for detailed visualization of ovarian architecture in the assessment of polycystic ovarian morphology.

**Figure 1:**
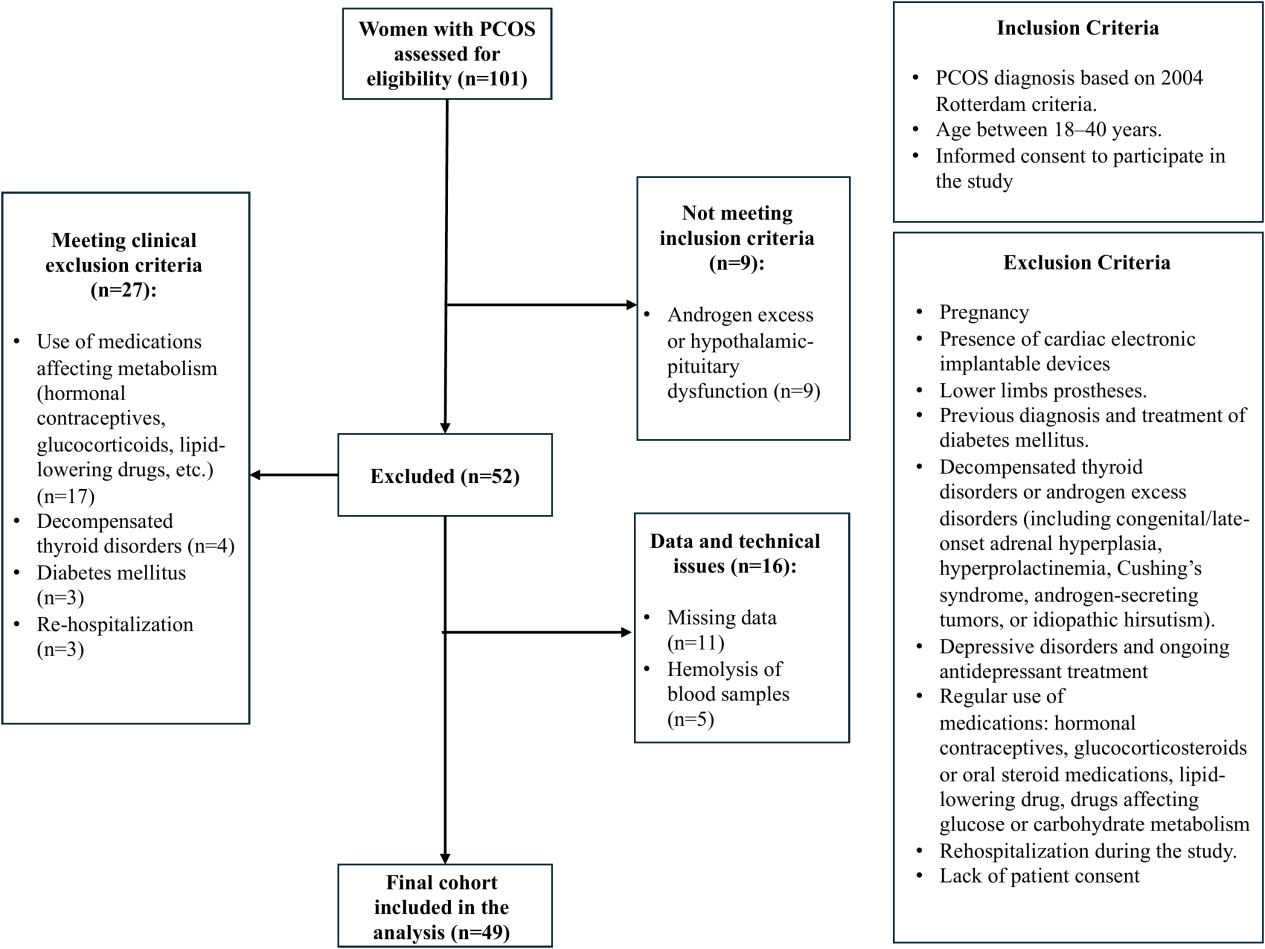
Recruitment process, inclusion and exclusion criteria.

A total of 101 women were initially screened for eligibility. Following the exclusion of individuals who did not fulfil the study criteria or presented additional disqualifying factors, 49 participants were ultimately enrolled. The recruitment flow, with the inclusion and exclusion criteria are presents on **Figure 1**. .

### 2.2 Diagnostic criteria

Diagnostic criteria for carbohydrate metabolism disturbances were defined as follows: impaired fasting glucose (100–125 mg/dL), impaired glucose tolerance (plasma glucose concentration of 140–199 mg/dL at 120. min of the OGTT) (26, 27) and insulin resistance, determined by a threshold HOMA-IR value ≥2.1 (27). Diabetes mellitus was diagnosed if at least one of the following criteria was met: the presence of symptoms of hyperglycaemia with a random plasma glucose ≥200 mg/dL, two separate fasting plasma glucose measurements ≥126 mg/dL, plasma glucose concentration ≥200 mg/dL at 120. min of the OGTT (26).

All procedures were carried out in line with the ethical standards set forth in the Declaration of Helsinki. The research protocol was reviewed and approved by the local bioethics committee (KNW/0022/KB1/143/15), and informed consent for participation and data analysis was obtained from every subject.

### 2.3 Data collection

Research was carried out at the Endocrinology Unit of Piekary Medical Centre, located within St. Luke’s Municipal Hospital in Piekary Śląskie, Poland. Blood samples were collected during morning hours, prior to any food intake. A one-millilitre aliquot of blood, initially collected for standard clinical evaluations, was retained for subsequent analysis. Following centrifugation, these samples were cryopreserved at -70 °C until the required assays were performed. All laboratory analyses were carried out in accordance with the Standard Work Instructions (SWI) at KORLAB Medical Laboratories. Plasma glucose was determined with a reference enzymatic hexokinase method on the Cobas Integra 400+ automated analyzer. Glycated haemoglobin (HbA1c) was assessed by high-performance liquid chromatography (HPLC) employing reversed-phase cation-exchange chromatography, using the ADAMS A1c HA-8180 analyzer. Fasting insulin levels were measured with the ARCHITECT insulin assay, a one-step immunoassay based on chemiluminescent microparticle technology with a microparticle tracer, enabling sensitive detection of human insulin in plasma or serum. Serum testosterone concentrations were quantified using a chemiluminescent microparticle immunoassay (CMIA) on the Abbott Alinity platform. Total cholesterol concentrations were measured using the Cobas Integra 400+ analyzer, employing an enzymatic colorimetric method. LDL cholesterol (LDL-C) was directly quantified with the MULTIGENT Direct LDL assay, which enables accurate measurement of LDL-C in serum or plasma without prior sample pretreatment or centrifugation, using the Architect c8000 analyzer. HDL cholesterol (HDL-C) concentrations were determined via a homogeneous enzymatic colorimetric technique on the Cobas Integra 400+ analyzer. Triglyceride levels were assessed using an enzymatic colorimetric method based on glycerol phosphate oxidase and 4-aminophenazone, also on the Cobas Integra 400+ platform.

The following biochemical parameters were evaluated: fasting plasma glucose, insulin, glycated haemoglobin A1c (HbA1c), total cholesterol (TC), high-density lipoprotein (HDL), low-density lipoprotein (LDL), and triglycerides (TG). These measurements were subsequently used for the calculation of relevant indices (**Table 1**).

**Table 1:** Anthropometric indices (25, 28–31).

Anthropometric indices	
BMI	body mass [kg] / height [m]2
WHR	waist circumference [cm] / hip circumference [cm]
WHtR	waist circumference [cm] / height [cm]
BAI	hip circumference [cm] / height [m]3/2-18
VAI *women*	[waist circumference [cm] / 36, 58+(1, 89xBMI [kg/m2))] x (TG [mmol/L] /0, 81) x (1, 52/HDL [mmol/L])
VAI *man*	[waist circumference [cm] / 39, 68+(1, 88xBMI [kg/m2))] x (TG [mmol/L] /1, 03) x (1, 31/HDL [mmol/L])
LAP *women*	[waist circumference [cm]-58) x TG [mmol/L]
LAP *man*	[waist circumference [cm]-65) x TG [mmol/L]
ABSI *women*	[waist circumference [m] / [BMI [kg/m2)3/5 x (height [m]1/5]
ABSI *man*	[waist circumference [m] / [BMI [kg/m2)2/3 x (height [m]1/2]
BRI	365.2−365.5 × √(1-(((WC/2π)2) / [(0.5 × height [m])]2)

BMI, Body Mass Index; WHR, Waist to Hip Ratio; WHtR, Waist to Height Ratio; BAI, Body Adiposity Index; VAI, Visceral Adiposity Index; LAP, Lipid Accumulation Product; ABSI, A Body Shape Index; BRI, Body Roundness Index; TG, Triglyceride.

Standard anthropometric measurements, including body weight [kg], height [cm], waist circumference [cm] (WC), and hip circumference [cm] (HC), were obtained in the morning, following an overnight fast. Participants were lightly clothed and barefoot during all assessments. All procedures adhered to standardized protocols, and calibrated, validated instruments were employed. Body weight was recorded to the nearest 0.01 kg using the Tanita BC 420 S MA Body Composition Analyzer (MDD93/42 EEC certified; Tanita Corp., Tokyo, Japan). Height was measured to the nearest 0.05 cm using the Tanita HR100 Stadiometer (Tanita Corp., Tokyo, Japan). Circumferences of the waist, hips, calves, and upper arms were determined to the nearest 1 mm with a SECA 203 measuring tape (Seca, Hamburg, Germany).

From the acquired measurements, individual anthropometric indices were computed and are summarized in **Table 1**.

Homeostatic model assessment (HOMA-IR) index was calculated using the formula: HOMA-IR = fasting insulinemia (mU/mL) x fasting glycemia (mmol/L)/22.5 (27). Insulin resistance was defined as HOMA-IR ≥2.1 (27), consistent with the criteria applied in the Study participant’s section.

Cut-off values and standards of the tested measurements and anthropometric measurements are presented in **Table 2**.

**Table 2:** Anthropometric measurements, reference standards, and corresponding health risk interpretations.

Tested measurements and anthropometric indicators	Standards	Interpretation of standards	References
WC (cm)	> 88 cm	significantly increased risk of metabolic complications	(25)
BMI Index (kg/m^2^)	< 18, 5 kg/m2	underweight	(28)
	18, 5-24, 9 kg/m2	normal body weight	
	25, 0-29, 9 kg/m2	overweight	
	30, 0-34, 9 kg/m2	obesity I	
	35, 0-39, 9 kg/m2	obesity II	
	≥ 40, 0 kg/m2	obesity III (enormous)	
WHR	≥0, 85	increasing the risk of metabolic complications	(25)
WHtR	≥0, 5	abdominal obesity	(25)
		increased risk cardiovascular diseases and diabetes	
BAI (%)	<21%	underweight	(32)
	21-33%	standard	
	>33%	overweight	
	>39%	obesity	
VAI	>1.675	risk of metabolic diseases	(29)
LAP	<41.30	discrimination of prediabetes/diabetes	(31)
BRI	<4.910	risk of metabolic diseases	(31)
ABSI	<0.076	risk of diabetes and cardiovascular diseases	(30, 31)

WC, waist circumference; BMI, body mass index; WHR, waist to hip ratio; WHtR, waist to height ratio; BAI, body adiposity index; VAI, visceral adiposity index; LAP, lipid accumulation product; BRI, body roundness index; ABSI, a body shape index.

Among the studied women, 26 had a WC greater than 88 cm, which may lead to significantly increased risk of metabolic complications. Excess body mass, defined as a BMI >25 was observed in 25 women. Moreover, 22 women with PCOS presented an increased risk of metabolic complication based on WHR index ≥0, 85. A total of 30 participants had a WHtR above 0.50, a threshold that correlates with elevated cardiometabolic risk. Excess body fat assessed using the BAI index was identified in 25 women. VAI >1.675 had 17 participants, which is associated with a risk of metabolic diseases. Based on the LAP index (<41.30), 27 women were classified as having prediabetes or diabetes. 28 participants had a BRI below 4.910. Finally, 21 women had an ABSI <0.076, which is linked to an elevated CVR and diabetes.

### 2.4 Statistical analysis

Statistical analyses were performed using PS IMAGO PRO 10 (IBM SPSS Statistics 29) and MedCalc^®^ version 23.3.7. Data distribution was assessed with the Shapiro-Wilk test. Continuous variables are presented as means ± standard deviations for normally distributed data or as medians with interquartile ranges (Q1–Q3) for non-normally distributed data. Group comparisons were conducted using Student’s t-test for normally distributed variables and the Mann-Whitney U test for non-normal variables. Correlations between continuous variables were evaluated using Pearson’s correlation coefficient for normally distributed data and Spearman’s rank correlation coefficient for non-normally distributed data.

The ability of selected anthropometric measures to predict insulin resistance risk (as assessed by HOMA-IR) was evaluated using Receiver Operating Characteristic (ROC) curve analysis. The area under the ROC curve (AUC) was calculated to quantify overall diagnostic performance, and the Youden index was applied to determine optimal cut-off values. Sensitivity, specificity, positive predictive value (PPV), and negative predictive value (NPV) were also calculated. Logistic regression models were employed to examine the association between individual anthropometric indices and the presence of insulin resistance. Odds ratios (ORs) with 95% confidence intervals (CIs) were reported. ROC analysis provided information on the discriminative ability of anthropometric parameters and their optimal thresholds for identifying individuals at risk of insulin resistance, whereas logistic regression quantified the strength of the association between these indices and insulin resistance risk. A p-value < 0.05 was considered statistically significant in all analyses.

## 3 Results

### 3.1 Characteristic of the study group

**Table 3** presents the anthropometric, metabolic, and hormonal parameters of women with PCOS. The median age was 25 years (22–29). The mean BMI was 28.3 ± 7.1 kg/m², BAI was 33 ± 5.7%. The mean fasting glucose was 5 ± 0.7 mmol/L, fasting insulin was 102.1 ± 64.1 pmol/L, median HOMA-IR was 2.7 (1.4–4.1). Insulin after 60 min in OGTT was 703.2 pmol/L (325–835.2), and mean glucose after 120 min in OGTT was 6.8 ± 2.1 mmol/L.

**Table 3:** Characteristics of anthropometric, metabolic, and hormonal parameters in women with polycystic ovary syndrome (PCOS).

Parameter	N	Total group mean ± SD me (Q1-Q3)
Age (years)	49	25 (22-29)
BMI (kg/m^2^)	49	28.3 ± 7.1
BAI (%)	49	33 ± 5.7
ABSI	49	0.1 ± 0.01
Waist circumference (cm)	49	91.1 ± 18.9
WHR	49	0.8 ± 0.1
WHtR	49	0.6 ± 0.1
Fasting glucose (mmol/l)	49	5 ± 0.7
Fasting insulin (pmol/L)	45	102.1 ± 64.1
HOMA-IR index	49	2.7 (1.4-4.1)
Insulin after 60 min (glucose tolerance test) (pmol/L)	36	703.2 (325-835.2)
Glucose after 120 min (glucose tolerance test) (mmol/L)	40	6.8 ± 2.1

SD, standard deviation; Me, median; Q1, 25. Percentyl; Q3, 75. Percentyl; BMI, Body Mass Index; BAI, Body Adiposity Index; ABSI, A Body Shape Index; WC, Waist Circumference; WHR, Waist-to-Hip Ratio; WHtR, Waist-to-Height Ratio; HOMA-IR, Homeostatic Model Assessment for Insulin Resistance.

Women with excess weight (overweight and obesity, based on BMI) showed significantly higher fasting insulin levels compared to women with normal weight 96.86 (69.60–137.40) vs. 48.07 (35.88–73.19) pmol/L, p<0.01. Fasting glucose was also higher in the overweight group 5.27 (4.83–5.47 vs. 4.66 (4.44–4.83) mmol/L, p<0.01. During the oral glucose tolerance test, women with excess weight had higher glucose levels after 60 minutes 9.00 (6.97–9.57) vs. 5.77 (4.61–8.38) mmol/L, p<0.01. The HOMA-IR index was significantly higher in the excess weight group 3.40 (2.20–5.15) vs. 1.24 (0.90–2.06), p<0.01, indicating greater insulin resistance.

Women with excess weight (based on BAI) had significantly higher fasting insulin 102.60 (72.11–137.40) vs. 50.94 (34.80–79.11) pmol/L, p<0.01, fasting glucose 5.27 (4.94–5.47) vs. 4.66 (4.44–4.84) mmol/L, p<0.01, and HOMA-IR 4.00 (2.20–5.15) vs. 1.55 (0.90–2.68), p<0.01compared to women with normal weight. Glucose after 60 minutes in OGTT was also higher 9.00 (6.77–9.68) vs. 7.27 (5.38–8.94) mmol/L, p=0.05. These findings are summarized in **Table 4**.

**Table 4:** Comparison of metabolic parameters between normal weight and excess weight women with PCOS based on BMI and BAI.

Parameter	N	Normal weight (based on BMI index)	N	Excess weight (overweight + obesity) (based on BMI index)	P value
Fasting insulin (pmol/L)	**17**	**48.07 (35.88-73.19)**	**31**	**96.86 (69.60-137.40)**	**p<0.01**
Fasting glucose (mmol/L)	**17**	**4.66 (4.44-4.83)**	**31**	**5.27 (4.83-5.47)**	**p<0.01**
Glucose after 120 min (mmol/L) (glucose tolerance test)	12	5.66 (4.63-6.74)	28	6.68 (5.63-8.35)	0.07
Glucose after 60 min (mmol/L) (glucose tolerance test)	**11**	**5.77 (4.61-8.38)**	**28**	**9.00 (6.97-9.57)**	**p<0.01**
Insulin after 60 min (pmol/L) (glucose tolerance test)	9	270.50 (229.60-830.87)	27	710.33 (528.08-892.57)	0.08
HOMA-IR index	**17**	**1.24 (0.90-2.06)**	**31**	**3.40 (2.20-5.15)**	**p<0.01**
	**Normal weight** **(based on BAI index)**		**Excess weight** **(overweight + obesity)** **(based on BAI index)**		
Fasting insulin (pmol/L)	**24**	**50.94 (34.80-79.11)**	**25**	**102.60 (72.11-137.40)**	**p<0.01**
Fasting glucose (mmol/L)	**24**	**4.66 (4.44-4.84)**	**25**	**5.27 (4.94-5.47)**	**p<0.01**
Glucose after 120 min (mmol/L) (glucose tolerance test)	16	6.24 (5.30-6.74)	24	6.73 (5.36-8.35)	0.19
Glucose after 60 min (mmol/L) (glucose tolerance test)	**15**	**7.27 (5.38-8.94)**	**24**	**9.00 (6.77-9.68)**	**0.05**
Insulin after 60 min (pmol/L) (glucose tolerance test)	13	340.81 (248.26-764.86)	23	710.33 (528.08-978.67)	0.06
HOMA-IR index	**24**	**1.55 (0.90-2.68)**	**25**	**4.00 (2.20-5.15)**	**p<0.01**

HOMA-IR, homeostatic model assessment; BMI, body mass index; BAI, body adiposity index.

Bold values indicate statistical significance (p < 0.05).

Women with waist circumference >88 cm had significantly higher fasting insulin 104.04 vs. 48.07 pmol/L, p<0.01, fasting glucose 5.24 vs. 4.72 mmol/L, p*=*0.01 and HOMA-IR 4.03 vs. 1.40, p < 0.01 compared to women with WC ≤88 cm. During OGTT, glucose at 60 minutes 9.05 vs. 5.88 mmol/L, p<0.01and insulin at 60 minutes 764.86 vs. 325.03 pmol/L, p*=*0.0225 were also higher in the group with elevated WC (**Table 5**).

**Table 5:** Comparison of metabolic parameters between normal weight and excess weight women with PCOS based on WC.

Parameter	WC ≤88 cm		WC >88 cm		
Fasting insulin (pmol/L)	**23**	**48.07 (33.72-73.19)**	**26**	**104.04 (86.46-144.22)**	**p<0.01**
Fasting glucose (mmol/L)	**23**	**4.72 (4.44-5.27)**	**26**	**5.24 (4.83-5.49)**	**0.01**
Glucose after 120 min (mmol/L) (glucose tolerance test)	15	5.66 (4.61-6.77)	25	6.69 (5.77-8.10)	0.08
Glucose after 60 min (mmol/L) (glucose tolerance test)	**14**	**5.88 (4.61-8.38)**	**25**	**9.05 (7.60-9.49)**	**p<0.01**
Insulin after 60 min (pmol/L) (glucose tolerance test)	**12**	**325.03 (238.93-756.25)**	**24**	**764.86 (536.69-935.62)**	**0.02**
HOMA-IR index	**23**	**1.40 (0.90-2.20)**	**26**	**4.03 (2.71-5.70)**	**p<0.01**

HOMA-IR, homeostatic model assessment; WC, waist circumference.

Bold values indicate statistical significance (p < 0.05).

Women with WHR ≥0.85 exhibited significantly elevated fasting insulin 104.04 vs. 52.38 pmol/L, p<0.01, fasting glucose 5.30 vs. 4.77 mmol/L, p=0.02, glucose at 120 minutes during OGTT 6.77 vs. 5.66 mmol/L, p=0.02, glucose at 60 minutes during OGTT 9.16 vs. 6.40 mmol/L, p<0.01, insulin at 60 minutes during OGTT 768.44 vs. 373.46 pmol/L, p=0.04, and HOMA-IR 4.03 vs. 1.70, p<0.01compared to women with WHR<0.85.

Similarly, patients with WHtR≥0.5 showed significantly higher fasting insulin 100.64 vs. 44.49 pmol/L, p<0.01, fasting glucose 5.27 vs. 4.72 mmol/L, p<0.01, glucose at 120 minutes during OGTT 6.73 vs. 5.66 mmol/L, p<0.01, glucose at 60 minutes during OGTT 9.07 vs. 5.55 mmol/L, p<0.01, insulin at 60 minutes during OGTT 764.86 vs. 297.05 pmol/L, p<0.01, and HOMA-IR 3.50 vs. 1.18, p<0.01 compared to women with WHtR<0.5 (**Table 6**).

**Table 6:** Comparison of metabolic parameters between normal weight and excess weight women with PCOS based on WHR and WHtR.

Parameter	WHR <0.85		WHR ≥0.85		
Fasting insulin (pmol/L)	27	52.38 (35.88-81.08)	22	104.04 (86.46-137.40)	p<0.01
Fasting glucose (mmol/L)	27	4.77 (4.44-5.27)	22	5.30 (4.83-5.72)	0.02
Glucose after 120 min (mmol/L) (glucose tolerance test)	19	5.66 (4.66-6.76)	21	6.77 (6.05-8.60)	0.02
Glucose after 60 min (mmol/L) (glucose tolerance test)	18	6.40 (5.38-7.66)	21	9.16 (7.99-9.66)	p<0.01
Insulin after 60 min (pmol/L) (glucose tolerance test)	16	373.46 (259.38-763.78)	20	768.44 (577.59-935.62)	0.04
HOMA-IR index	27	1.70 (0.90-3.05)	22	4.03 (2.71-5.15)	p<0.01
		WHtR <0.5		WHtR ≥0.5	
Fasting insulin (pmol/L)	19	44.49 (32.29-69.60)	30	100.64 (77.13-137.40)	p<0.01
Fasting glucose (mmol/L)	19	4.72 (4.44-4.83)	30	5.27 (4.83-5.49)	p<0.01
Glucose after 120 min (mmol/L) (glucose tolerance test)	12	5.66 (4.52-6.49)	28	6.73 (5.63-8.35)	p<0.01
Glucose after 60 min (mmol/L) (glucose tolerance test)	11	5.55 (4.44-6.53)	28	9.07 (7.69-9.68)	p<0.01
Insulin after 60 min (pmol/L) (glucose tolerance test)	10	297.05 (229.60-681.63)	26	764.86 (529.52-978.67)	p<0.01
HOMA-IR index	19	1.18 (0.78-2.06)	30	3.50 (2.65-5.15)	p<0.01

HOMA-IR, homeostatic model assessment; WHR, waist to hip ratio; WHtR, waist to height ratio.

Patients with VAI>1.675 showed significantly higher fasting insulin 98.68 vs. 59.73 pmol/L, p<0.01, glucose after 120 minutes during OGTT 6.99 vs. 6.22 mmol/L, p=0.04, glucose after 60 minutes during OGTT 9.05 vs. 7.63 mmol/L, p=0.04, and HOMA-IR index 3.23 vs. 1.77, p<0.01compared to women with VAI ≤ 1.675.

Women with LAP≥41.30 had significantly higher fasting insulin 113.19 vs. 49.51 pmol/L, p<0.01, fasting glucose 5.30 vs. 4.72 mmol/L, p<0.01, glucose after 60 minutes during OGTT 9.10 vs. 6.90 mmol/L, p<0.01, insulin after 60 minutes during OGTT 768.44 vs. 332.20 pmol/L, p=0.0162, and HOMA-IR index 4.03 vs. 1.70, p<0.01 compared to women with LAP<41.30 (**Table 7**).

**Table 7:** Comparison of metabolic parameters between normal weight and excess weight women with PCOS based on VAI and LAP.

Parameter	VAI ≤1.675		VAI >1.675		
Fasting insulin (pmol/L)	**32**	**59.73 (36.06-95.79)**	**17**	**98.68 (86.46-137.40)**	**p<0.01**
Fasting glucose (mmol/L)	32	4.83 (4.55-5.36)	17	5.22 (4.83-5.38)	0.24
Glucose after 120 min (mmol/L) (glucose tolerance test)	**25**	**6.22 (5.11-6.76)**	**15**	**6.99 (5.83-9.27)**	**0.04**
Glucose after 60 min (mmol/L) (glucose tolerance test)	**24**	**7.63 (5.63-9.13)**	**15**	**9.05 (7.10-10.55)**	**0.04**
Insulin after 60 min (pmol/L) (glucose tolerance test)	23	681.63 (270.50-830.87)	13	764.86 (528.08-1051.86)	0.21
HOMA-IR index	**32**	**1.77 (0.99-3.83)**	**17**	**3.23 (3.05-5.15)**	**p<0.01**
	LAP <41.30		LAP ≥41.30		
Fasting insulin (pmol/L)	**27**	**49.51 (35.88-76.77)**	**22**	**113.19 (92.20-144.22)**	**p<0.01**
Fasting glucose (mmol/L)	**27**	**4.72 (4.44-5.27)**	**22**	**5.30 (4.94-5.47)**	**p<0.01**
Glucose after 120 min (mmol/L) (glucose tolerance test)	19	6.22 (4.66-6.77)	21	6.76 (5.77-8.60)	0.06
Glucose after 60 min (mmol/L) (glucose tolerance test)	**18**	**6.90 (5.38-8.66)**	**21**	**9.10 (7.10-9.71)**	**p<0.01**
Insulin after 60 min (pmol/L) (glucose tolerance test)	**16**	**332.20 (255.43-797.86)**	**20**	**768.44 (577.59-1015.26)**	**0.02**
HOMA-IR index	**27**	**1.70 (0.90-2.20)**	**22**	**4.03 (3.10-5.70)**	**p<0.01**

HOMA-IR, homeostatic model assessment; VAI, visceral adiposity index; LAP, lipid accumulation product.

Bold values indicate statistical significance (p < 0.05).

Patients with BRI≥4.910 showed significantly higher fasting insulin 123.77 vs. 50.94 pmol/L, p<0.01, fasting glucose 5.33 vs. 4.72 mmol/L, p<0.01, glucose after 120 minutes during OGTT 6.88 vs. 5.72 mmol/L, p=0.01, glucose after 60 minutes during OGTT 9.13 vs. 6.72 mmol/L, p<0.01, insulin after 60 minutes during OGTT 772.03 vs. 340.81 pmol/L, p=0.02, and HOMA-IR index 4.08 vs. 1.70, p<0.01 compared to women with BRI<4.910.

Women with ABSI≥0.076 had significantly higher fasting insulin 83.95 vs. 49.51 pmol/L, p=0.0102, and glucose after 60 minutes during OGTT 9.13 vs. 5.99 mmol/L, p<0.01compared to women with ABSI<0.076 (**Table 8**).

**Table 8:** Comparison of metabolic parameters between normal weight and excess weight women with PCOS based on BRI and ABSI.

Parameter	BRI <4.910		BRI ≥4.910		
Fasting insulin (pmol/L)	**28**	**50.94 (35.88-76.95)**	**21**	**123.77 (92.20-144.22)**	**p<0.01**
Fasting glucose (mmol/L)	**28**	**4.72 (4.50-5.24)**	**21**	**5.33 (4.94-5.72)**	**p<0.01**
Glucose after 120 min (mmol/L) (glucose tolerance test)	**20**	**5.72 (4.86-6.60)**	**20**	**6.88 (5.94-8.92)**	**0.01**
Glucose after 60 min (mmol/L) (glucose tolerance test)	**19**	**6.72 (5.38-8.38)**	**20**	**9.13 (7.94 -9.68)**	**p<0.01**
Insulin after 60 min (pmol/L) (glucose tolerance test)	**17**	**340.81 (262.61-764.86)**	**19**	**772.03 (625.66-978.67)**	**0.02**
HOMA-IR index	**28**	**1.70 (0.90-2.68)**	**21**	**4.08 (3.20-5.70)**	**p<0.01**
	ABSI <0.076		ABSI ≥0.076		
Fasting insulin (pmol/L)	**15**	**49.51 (40.90-80.36)**	**21**	**83.95 (60.99-116.24)**	**0.01**
Fasting glucose (mmol/L)	21	4.83 (4.66-5.27)	28	5.22 (4.58-5.56)	0.21
Glucose after 120 min (mmol/L) (glucose tolerance test)	16	5.99 (4.88-6.77)	24	6.68 (5.58-8.35)	0.14
Glucose after 60 min (mmol/L) (glucose tolerance test)	**15**	**5.99 (5.38-7.10)**	**24**	**9.13 (7.94-9.68)**	**p<0.01**
Insulin after 60 min (pmol/L) (glucose tolerance test)	14	511.22 (270.50-830.87)	22	737.59 (528.08-892.57)	0.23
HOMA-IR index	21	2.06 (0.90-3.20)	28	3.10 (1.85-4.53)	0.06

HOMA-IR, homeostatic model assessment; BRI, body roundness index; ABSI, A body shape index.

Bold values indicate statistical significance (p < 0.05).

Patients with BRI≥4.910 showed significantly higher fasting insulin 123.77 vs. 50.94 pmol/L, p<0.01, fasting glucose 5.33 vs. 4.72 mmol/L, p<0.01, glucose after 120 minutes during OGTT 6.88 vs. 5.72 mmol/L, p=0.01, glucose after 60 minutes during OGTT 9.13 vs. 6.72 mmol/L, p<0.01, insulin after 60 minutes during OGTT 772.03 vs. 340.81 pmol/L, p=0.02, and HOMA-IR index 4.08 vs. 1.70, p<0.01 compared to women with BRI<4.910.

Women with ABSI≥0.076 had significantly higher fasting insulin 83.95 vs. 49.51 pmol/L, p=0.0102, and glucose after 60 minutes during OGTT 9.13 vs. 5.99 mmol/L, p<0.01compared to women with ABSI<0.076 (**Table 8**).

**Figure 2:**
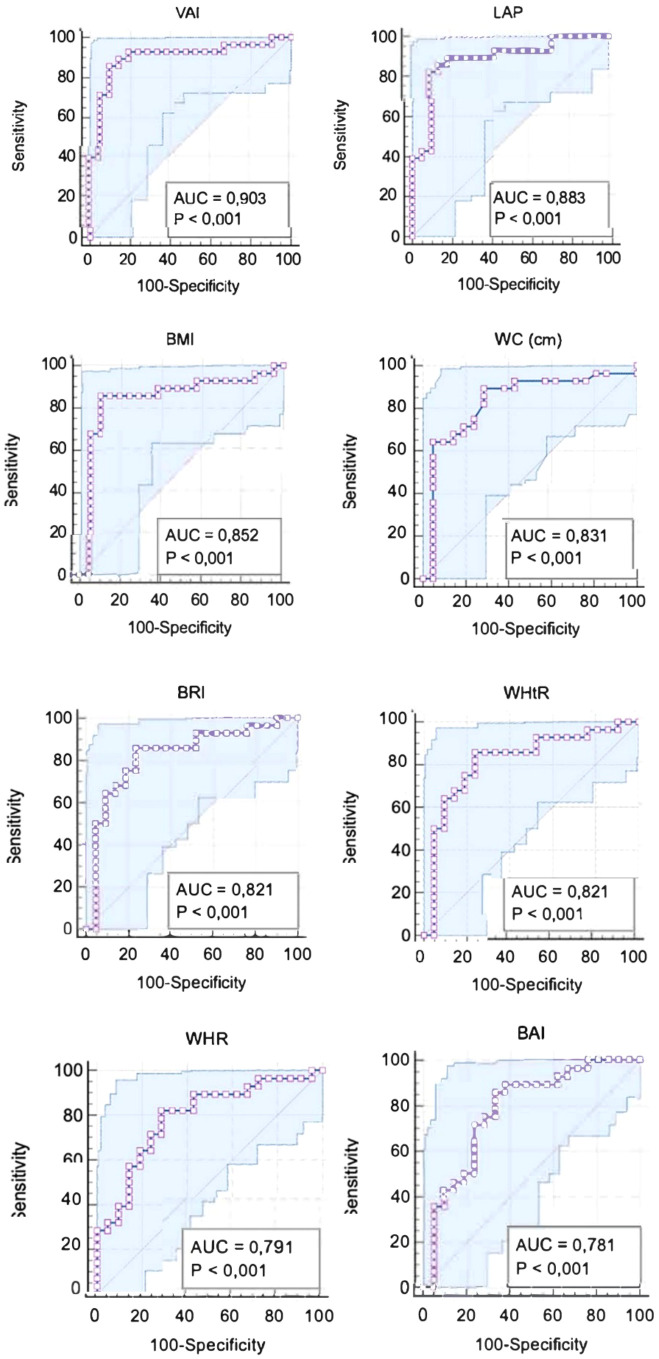
Receiver-operating characteristic (ROC) curves identifying the threshold for HOMA-IR for VAI, LAP, BMI, WC, BRI, WHtR, WHR, BAI.

**Table 9** shows the diagnostic performance of selected anthropometric and metabolic indices in predicting glucose and insulin dysregulation in women with PCOS. The analysis of anthropometric parameters and metabolic indices in relation to HOMA-IR values showed that most of the tested indicators had high predictive ability, with notable differences in performance. The VAI yielded the best results, with the highest area under the ROC curve (AUC = 0.903; 95% CI: 0.78–0.97), indicating excellent discriminatory ability. The optimal cut-off value was 0.99, with a sensitivity of 85.7%, specificity of 90.5%, positive predictive value (PPV) of 92.3%, and negative predictive value (NPV) of 82.6%, suggesting VAI is a particularly effective indicator for identifying elevated HOMA-IR levels. LAP was the second most effective indicator, with an AUC of 0.883 (95% CI: 0.76–0.96) and a cut-off of 27.9, demonstrating a sensitivity of 82.1%, PPV of 92%, and NPV of 79.2%. BMI also showed strong predictive value (AUC = 0.852; 95% CI: 0.72–0.94), with a cut-off of 27 kg/m², sensitivity of 85.7%, specificity of 90.5%, and PPV of 92.3%, highlighting its utility as a predictor of insulin resistance. Both waist-to-height ratio (WHtR) and waist circumference (WC) exhibited high AUCs (0.821 and 0.831, respectively) with sensitivities of 85.7% and 89.3%, reflecting good predictive performance. WHR achieved an AUC of 0.791, with a sensitivity of 82.1%, specificity of 71.4%, and NPV of 75%, indicating moderate effectiveness. The Body Adiposity Index (BAI) also performed well (AUC = 0.781), with a cut-off of 31.5%, sensitivity of 85.7%, and specificity of 66.7%. Body Roundness Index (BRI) had an AUC of 0.821, comparable to WHtR, whereas A Body Shape Index (ABSI) showed limited utility with a lower AUC of 0.643. Receiver-operating characteristic (ROC) curves identifying the threshold for HOMA-IR for VAI, LAP, BMI, WC, BRI, WHtR, WHR, BAI are presented on **Figure 2**.

**Table 9:** Predictive value of anthropometric and metabolic parameters for glycaemic and insulinemic markers.

Parameters	Parameters cut-off	AUC (95% CI)	Sensitivity (%)	Specificity (%)	PPV (%)	NPV (%)
Fasting glucose (mmol/L) Positive group:9; negative group:40; no data: 0						
WC (cm)	90	0.707 (0.56-0.83)	77.8	60	30.4	92.3
BMI (kg/m^2^)	33.6	0.678 (0.53-0.80)	55.6	82.5	41.7	89.2
WHR	0.84	0.719 (0.57-0.84)	77.8	62.5	31.8	92.6
WHtR	0.6	0.686 (0.54-0.81)	77.8	62.5	31.8	92.6
BAI (%)	35.6	0.644 (0.50-0.78)	55.6	77.5	35.7	88.6
VAI	1.41	0.503 (0.36-0.65)	77.8	42.5	23.3	89.5
LAP	16.9	0.6 (0.45-0.74)	88.9	42.5	25.8	94.4
BRI	4.5	0.686 (0.54-0.81)	77.8	65	33.3	92.9
ABSI	0.08	0.644 (0.50-0.78)	77.8	60	30.4	92.3
Fasting insuline (pmol/L) Positive group:16; negative group:29; no data: 4						
WC (cm)	101	0.811 (0.67-0.91)	81.3	89.7	81.2	89.7
BMI (kg/m^2^)	29.1	0.821 (0.68-0.92)	87.5	82.8	73.7	92.3
WHR	0.87	0.780 (0.63-0.89)	75	75.9	63.2	84.6
WHtR	0.63	0.810 (0.67-0.91)	75	89.7	80	86.7
BAI (%)	32.46	0.749 (0.60-0.87)	87.5	55.2	51.9	88.9
VAI	0.96	0.683 (0.53-0.81)	93.8	58.6	55.6	94.4
LAP	27.9	0.737 (0.59-0.86)	87.5	62.1	56	90
BRI	6.06	0.810 (0.67-0.91)	75	89.7	80	86.7
ABSI	0.08	0.629 (0.47-0.77)	68.8	62.1	50	78.3
HOMA-IR Positive group:28; negative group:11; no data: 0						
WC (cm)	76	0.831 (0.7-0.92)	89.3	71.4	80.6	83.3
BMI (kg/m^2^)	27	0.852 (0.72-0.94)	85.7	90.5	92.3	82.6
WHR	0.79	0.791 (0.65-0.89)	82.1	71.4	79.3	75
WHtR	0.5	0.821 (0.69-0.92)	85.7	76.2	82.8	80
BAI (%)	31.5	0.781 (0.64-0.89)	85.7	66.7	77.4	77.8
VAI	0.99	0.903 (0.78-0.97)	85.7	90.5	92.3	82.6
LAP	27.9	0.883 (0.76-0.96)	82.1	90.5	92	79.2
BRI	3.36	0.821 (0.69-0.92)	85.7	76.2	82.8	80
ABSI	0.08	0.643 (0.49-0.78)	60.7	71.4	73.9	57.7
Glucose after 120 min (mmol/L) (glucose tolerance test) Positive group:10; negative group:30; no data: 9						
WC (cm)	104	0.725 (0.56-0.85)	70	80	53.8	88.9
BMI (kg/m^2^)	27.5	0.7 (0.53-0.83)	90	60	42.9	94.7
WHR	0.87	0.733 (0.57-0.86)	80	66.7	44.4	90.9
WHtR	0.61	0.717 (0.55-0.85)	80	73.3	50	91.7
BAI (%)	32.8	0.603 (0.44-0.75)	80	46.7	33.3	87.5
VAI	0.99	0.703 (0.54-0.84)	90	53.3	39.1	94.1
LAP	72.7	0.753 (0.59-0.88)	60	86.7	60	86.7
BRI	5.7	0.717 (0.55-0.85)	80	73.3	50	91.7
ABSI	0.08	0.643 (0.48-0.79)	80	56.7	38.1	89.5
Insulin after 60 min (pmol/L) (glucose tolerance test) Positive group:25; negative group:11; no data: 13						
WC (cm)	87	0.782 (0.61-0.90)	84	72.7	87.5	66.7
BMI (kg/m^2^)	25.6	0.782 (0.61-0.90)	84	72.7	87.5	66.7
WHR	0.78	0.793 (0.63-0.91)	84	72.7	87.5	66.7
WHtR	0.52	0.775 (0.61-0.89)	84	72.7	87.5	66.7
BAI (%)	30.7	0.744 (0.57-0.87)	92	54.6	82.1	75
VAI	0.77	0.735 (0.56-0.87)	88	63.6	84.6	70
LAP	26	0.771 (0.60-0.89)	84	72.7	87.5	66.7
BRI	3.70	0.775 (0.61-0.89)	84	72.7	87.5	66.7
ABSI	0.08	0.709 (0.53-0.85)	72	72.7	85.7	53.3

BMI, Body Mass Index; BAI, Body Adiposity Index; ABSI, A Body Shape Index; WC, Waist Circumference; WHR, Waist-to-Hip Ratio; WHtR, Waist-to-Height Ratio; HOMA-IR, Homeostatic Model Assessment for Insulin Resistance.

### 3.2 Odds ratios for predicting insulin resistance in women with PCOS based on various anthropometric indices

In the studied group most of the examined anthropometric and metabolic indices were significantly associated with insulin resistance (**Table 10**). The strongest associations were observed for body mass index (BMI) and visceral adiposity index (VAI), both showing a 57-fold higher risk of insulin resistance (BMI≥27.25kg/m²: OR = 57.0; 95%CI:9.41–345.15; p<0.001; VAI≥1.07: OR = 57.0; 95%CI:9.41–345.15; p<0.001). Waist circumference (WC≥77.25cm: OR = 20.8; 95%CI:4.53–95.89; p<0.001), waist-to-height ratio (WHtR≥0.51: OR = 19.2; 95%CI:4.46–82.60; p<0.001), and lipid accumulation product (LAP≥32.04: OR = 43.7; 95%CI:7.60–251.17; p<0.001) also showed significant predictive value. Other significant predictors included waist-to-hip ratio (WHR≥0.79: OR = 11.5; 95%CI:2.97–44.51; p<0.001), body adiposity index (BAI≥31.5%: OR = 11.1; 95%CI:2.74–45.26; p=0.001), and body roundness index (BRI≥3.48: OR = 19.2; 95%CI:4.46–82.60; p<0.001.

**Table 10:** Odds ratios (with 95% confidence interval) in predicting of predicting insulin resistance in women with PCOS for various anthropometric indices.

Indices	Women group		
	OR	95% CI	p
WC (cm)	20.8	4.53-95.89	<0.001
BMI (kg/m^2^)	57.0	9.41-345.15	<0.001
WHR	11.5	2.97-44.51	<0.001
WHtR	19.2	4.46-82.60	<0.001
BAI (%)	11.1	2.74-45.26	0.001
VAI	57.0	9.41-345.15	<0.001
LAP	43.7	7.60-251.17	<0.001
BRI	19.2	4.46-82.60	<0.001
ABSI	3.2	0.92-11.15	0.06

## 4 Discussion

Considered the widespread occurrence of hyperinsulinemia in women with PCOS, our study specifically focused on assessing parameters of carbohydrate metabolism parameters in this group. IR is a key feature of PCOS; however, clinically available insulin estimation has limited accessibility and are not recommended for routine screening (14). Simple anthropometric indices provide a practical, non-invasive alternative for preliminary assessment of glucose dysregulation and IR. The effectiveness of anthropometric indices such as BMI, BAI, WHtR, ABSI, BRI in predicting CVD risk has also been confirmed in in non-European populations (33). However, data regarding the predictive value of these indices remain limited, underscoring the need for population specific cut-off points and further validation.

ROC analysis in the study were used to determine suggested cut-off values for IR. We identified a BMI cut-off point of 27.0 (AUC = 0.852) for detecting IR, which is similar to the value 27.1 (AUC = 0.745) reported by Hatami H et al. (34), but higher than the BMI cut-off point 23.05 (AUC = 0.799) reported by Zhu et al. (35). HOMA-IR cut-offs varied across studies (36) we applied a threshold of ≥2.1. These differences, along with variable cut off points for anthropometric measures like BMI, may partly explain discrepancies in correlations with IR. In present study, the WHR cut-off point was 0.79 (AUC = 0.791), compared to 0.852 (AUC = 0.690) (37) and 0.80 (AUC = 0.678) (34) reported by others. The WC cut-off point was 76 cm (AUC = 0.831), whereas other studies reported 84.9 cm (AUC = 0.786) (35) and 87.5 cm (AUC = 0.745) (34). WHtR performed well in classifying women with PCOS as having IR, with the optimal cut-off at 0.50 (AUC = 0.82), close to the values reported by Zhu et al. 0.519 (AUC = 0.777) (35) and 0.54 (AUC = 0.739) (34).

Hatami et al. (34) established a BAI cut-off value of 32.512 (AUC = 0.687) for IR, while our results found 31.5 (AUC = 0.781) to be optimal based on HOMA-IR. Similarly, ABSI value identified in our study was 0.08 (AUC = 0.643), comparable to the 0.075 (AUC = 0.567) cut-off suggested by previous authors (34).

VAI represents simple and practical tool in the assessment of metabolic disturbances among women with PCOS, as it incorporates BMI, WC, TG, and HDL-C concentration. The cut-off points for VAI based on HOMA-IR was 0.99 (AUC = 0.903). VAI and LAP demonstrated strong discriminatory power for identifying elevated fasting glucose and insulin levels, and HOMA-IR (AUC = 0.903, AUC = 0.883, respectively; p<0.001), highlighting cut-off points above which these metabolic disturbances are likely to occur. For LAP we established cut-off value of 27.9. Similar conclusions were reported by Naghsband Z et al. (37), who also highlighted VAI and LAP as useful tools for the initial screening cardiometabolic disturbances in women with PCOS. The superior performance of VAI and LAP suggests they capture fat quality rather than just fat mass. These indices reflect adipose dysfunction (adiposopathy) – marked by altered lipid fluxed and low-grade inflammation – which is central to PCOS pathogenesis even in non-obese individuals. This metabolic remodeling paradigm provides a more biologically plausible assessment of risk than BMI alone, consistent with modern cardiometabolic frameworks (38).

Among all tested parameters, VAI, LAP, and BMI showed the highest predictive ability for HOMA-IR, highlighting their value for identifying women at risk of IR, and for early detection of metabolic disorders related to glucose testing (36). Furthermore, another meta-analysis confirmed that both LAP and VAI are significantly her in PCOS women compared to healthy controls, further supporting their utility in glucose and insulin assessment (39). For BRI we found the discriminatory of AUC = 0.821 with cut-off point 3.36. In contrast, the ABSI index, which combined BMI, WC, height showed limited utility cut-off of 0.08 (AUC = 0.643).

Recent evidence indicates that WHtR exhibits the strongest association with excess androgen secretion (40) and IR (35) representing a sensitive screening marker (35, 40). A recent meta-analysis further confirmed that women with PCOS and IR have significantly higher WHtR compared to those without IR (36). Although WHtR strongly correlates with IR and may support early risk stratification in PCOS, based on the Rotterdam 2023 guidelines, abnormalities in glucose metabolism still require confirmation through glucose laboratory tests (14).

The VAI index has been demonstrated as a superior predictor of metabolic syndrome (MetS) in women with PCOS compared to other indices, including HOMA-IR and LAP (41, 42). Both VAI and LAP showed significant correlations with fasting glucose and insulin, confirming their utility as markers of IR and MetS in PCOS (37, 43). In contrast, ABSI demonstrated limited predictive value, emphasizing that not all anthropometric indices are equally informative for assessing metabolic risk in this population (44).

In our study, the HOMA-IR cut-off point was defined as ≥2.1, which was established using SHBG levels as a reference marker for insulin resistance in a large cohort of Caucasian women with PCOS (27). As we mentioned above, HOMA-IR cut-offs varied across studies, ranging from 2.0 to 3.8 (36). The one of limited study which also established cut-off points for women with PCOS due to IR ranged HOMA-IR ≥2.6 (34). Migacz M et al. (45) reported no significant differences in HOMA-IR values between different PCOS clinical outcomes suggesting that PCOS may not substantially influence screening for carbohydrate metabolism disturbances, and such assessments should be recommended regardless of clinical patterns in PCOS. Akkus et al. (46) demonstrated that VAI significantly correlates with the highest values observed in PCOS clinical outcomes characterized by hyperandrogenism and oligo-anovulation. This indicates that while HOMA-IR provides a global measure of IR applicable to all PCOS patients, VAI may offer additional granularity, allowing identification of women with a higher cardiometabolic risk (22). Moreover, while VAI remains elevated across most PCOS women, LAP appears more sensitive to BMI and may lose significance in lean patients. This suggests that VAI can be a reliable screening tool independent of body mass, whereas LAP may by more informative in overweight or obese women (39, 43). These observations align with studies emphasizing the importance of central and overall adiposity in mediating metabolic risk, underscoring the relevance of incorporating anthropometric indices into clinical assessment of women with PCOS (47).

Nonetheless, several limitations should be acknowledged. First, and foremost, although the study sample was carefully recruited based on strict inclusion criteria, the final sample size was relatively small (n=49). While this sample was sufficient to demonstrate statistical significance for the primary endpoints, the small cohort size may limit the statistical power for secondary analyses and external generalizability of the proposed cut-off values. Specifically, the high Odds Ratios observed for indices such as VAI and BMI were accompanied by wide confidence intervals. This suggests a potential overestimation of the effect size; a phenomenon often encountered in smaller cohorts with high-performing predictors. Therefore, while our results provide a strong signal regarding the utility of these markers, the specific cut-off points and magnitude of the OR should be interpreted as preliminary. These findings require validation in larger, independent cohorts before being implemented in routine clinical practice. Furthermore, IR was assessed using HOMA-IR rather than the hyperinsulinemic-euglycemic clamp due to the complexity of the latter (despite it being the gold standard). Finally, the cross-sectional design of the study precludes establishing causality between the anthropometric parameters and the onset of metabolic dysregulation.

Despite these limitations, our study supports the clinical utility of non-invasive anthropometric measures as practical, first-line screening tools for early detection of glucose dysregulation in women with PCOS. By proposing specific cut-off points, this research provide pragmatic instrument for preliminary metabolic risk stratification, particularly in settings where laboratory-based insulin measurments are not routinely available. This approach aligns with the evolving paradigm of personalized cardiometabolic prevention, identyfiying patients who may benefit from early lifestyle interventions or emerging targeted therapies (48). However, given the explorstory nature of these resukts and the small sample size, further validatin in larger, independent cohorts is essential to confirm the clinical reliability of these diagnostic threshold.

## 5 Conclusions

Anthropometric indices, including BMI, WHR, WHtR, BAI, VAI, LAP, ABSI and BRI demonstrate significant predictive value for glucose dysregulation, particularly IR, with VAI and BMI showing the strongest association. They may provide a practical, non-invasive and cost-effective approach for preliminary metabolic screening. Early identification of at-risk individuals using these indices may facilitate timely interventions to prevent cardiometabolic complications. Further studies with larger cohorts are recommended to validate optimal cut-off points and assed their predictive accuracy across diverse populations women with PCOS.

## Data Availability

The original contributions presented in the study are included in the article/supplementary material. Further inquiries can be directed to the corresponding author.
